# Group trust dynamics during a risky driving experience in a Tesla Model X

**DOI:** 10.3389/fpsyg.2023.1129369

**Published:** 2023-06-20

**Authors:** Ali Momen, Ewart J. de Visser, Marlena R. Fraune, Anna Madison, Matthew Rueben, Katrina Cooley, Chad C. Tossell

**Affiliations:** ^1^United States Air Force Academy, Colorado Springs, CO, United States; ^2^Department of Psychology, New Mexico State University, Las Cruces, NM, United States; ^3^United States Army Research Laboratory, Aberdeen Proving Ground, Aberdeen, MD, United States

**Keywords:** autonomous vehicles, automated driving systems, trust calibration, team mental models, group polarization, contagion, trust propagation, self-driving vehicle design features

## Abstract

The growing concern about the risk and safety of autonomous vehicles (AVs) has made it vital to understand driver trust and behavior when operating AVs. While research has uncovered human factors and design issues based on individual driver performance, there remains a lack of insight into how trust in automation evolves in groups of people who face risk and uncertainty while traveling in AVs. To this end, we conducted a naturalistic experiment with groups of participants who were encouraged to engage in conversation while riding a Tesla Model X on campus roads. Our methodology was uniquely suited to uncover these issues through naturalistic interaction by groups in the face of a risky driving context. Conversations were analyzed, revealing several themes pertaining to trust in automation: (1) collective risk perception, (2) experimenting with automation, (3) group sense-making, (4) human-automation interaction issues, and (5) benefits of automation. Our findings highlight the untested and experimental nature of AVs and confirm serious concerns about the safety and readiness of this technology for on-road use. The process of determining appropriate trust and reliance in AVs will therefore be essential for drivers and passengers to ensure the safe use of this experimental and continuously changing technology. Revealing insights into social group–vehicle interaction, our results speak to the potential dangers and ethical challenges with AVs as well as provide theoretical insights on group trust processes with advanced technology.

## 1. Introduction

### 1.1. Risks with self-driving vehicles and the need to evaluate trust

Autonomous vehicles (AVs) are transforming transportation systems via breakthroughs in technology including advanced sensors, artificial intelligence, and machine learning algorithms (Bonnefon et al., 2016; Fleetwood, 2017; Merat et al., 2019). In Pittsburgh, users can ride in the backseat of an autonomous Waymo with an employee who supervises the automation (Wayland, 2022). The University of Texas at Arlington offers its students rides around campus with fully automated public transit vehicles powered by the May Mobility technology (Bishop, 2023). In Shanghai and Beijing, autonomous vehicles by AutoX can transport people and goods on even the most challenging roads (AutoX Careers, Perks + Culture, 2023).

AVs have nonetheless been met with highly publicized setbacks. The NHTSA is investigating several fatal crashes associated with AVs (Man Behind Wheel in Tesla Autopilot Crash that Killed Two is Charged, 2022; Tesla Crash that Killed California Couple Investigated by NHTSA, 2022). In another recent example, a couple in a Honda Civic was killed after a Tesla exiting the highway ran a red light and crashed into them. The State of California is suing Tesla for misleading the public about the Tesla autopilot capabilities, and news reporters have flooded social media with first-person views of Tesla performing dangerously on city roads [CNN (Director), 2021]. This has raised concerns about the risk of riding in a partially automated or fully autonomous vehicle and whether drivers or riders of vehicles can trust such vehicles (Endsley, 2017; Molnar et al., 2018; Ekman et al., 2019; Kraus et al., 2020, 2021; Ayoub et al., 2022). Addressing such concerns will be paramount to public acceptance of AVs and to ensure its safe and successful integration into our traffic systems (Banks et al., 2018a).

### 1.2. Trust and perceptions of risk theory

**Figure 1:**
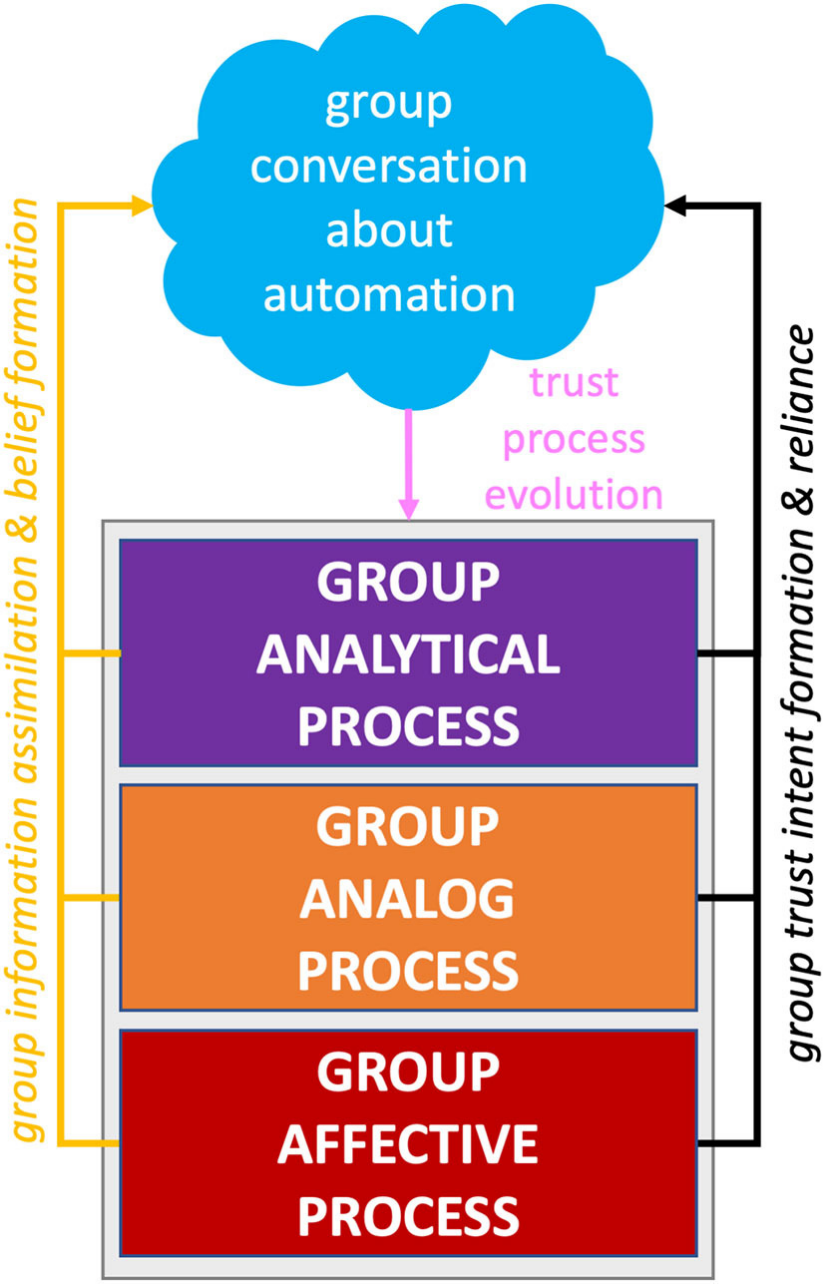
Hypothesized model of group trust processes based on Lee and See's (2004) Model.

Based on the article by Lee and See (2004), trust is defined as the confidence an individual has in another agent within a context that involves elements of risk and vulnerability. A recent study has delved into trust and performance in AVs in which the driving automation represents the trusted agent (Molnar et al., 2018; Ekman et al., 2019; Petersen et al., 2019; Lee et al., 2021; Ayoub et al., 2022). Banks et al. (2018b) found that drivers interacted with AVs in a way that indicated over-trust and complacency in the AV. Endsley (2017) provided insight into these issues by attributing them to a lack of transparency in the vehicle, such as in the case of mode confusion and a general lack of mental model development of the system's capabilities. These studies have demonstrated that trust evolves through an interplay of complementary processes (Lee and See, 2004; Hancock et al., 2021). Analytical processes involve cognitive reasoning and rational decision-making about interactions with another agent. Analog processes involve categorical judgments based on direct observation and reports from parties that have experience with the agent, group membership, norms and etiquette, team roles, reputation, and gossip. Finally, affective processes involve the rapid emotional responses that can arise when risk is involved when interacting and depending on another agent (Loewenstein et al., 2001; Slovic et al., 2007). Lee and See's (2004, p. 54) model further expands on the utilization of analytical, analog, and affective information in a four-stage model: (1) information assimilation and belief formation, (2) trust evolution, (3) intention formation, and (4) reliance action, which is reproduced in our model (see Figure 1). Due to the novelty of AVs, riders and drivers will likely use whatever information is available to them to assess associated risk, determine the trustworthiness of AVs, and then whether they themselves will trust AVs. Additionally, users will likely vary in their affective and individual risk tolerance and risk-taking behaviors (Kannadhasan, 2015). Furthermore, group trust dynamics expressed through conversation will likely shape exactly how users utilize information as the trust process evolves (Li et al., 2023). It is therefore important to put users in a situation where we can examine how these processes unfold together and assess how trust processes manifest themselves in the AV situation.

### 1.3. Real-world evaluation of trust and the value of constructive interaction in groups

The prominence of potential risk with self-driving vehicles highlights the importance of naturalistic experiments to uncover driver behavior in response to perceptions of risk (Bolstad and Endsley, 1999; Banks and Stanton, 2016; Banks et al., 2018a; Tenhundfeld et al., 2019, 2020; DSouza et al., 2021; Belcher et al., 2022). The resulting adjustment in behavior and trust attitudes observed with riders in response to perceptions of risk may not be induced in a simulator (Kohn et al., 2021; Krausman et al., 2022). Indeed, most driving research is conducted in driving simulators or controlled laboratories, which lack the risk and vulnerability associated with real traffic and potentially weaken ecological validity (Kemeny and Panerai, 2003; Santos et al., 2005). For assessments of trust, which involve situations of risk and vulnerability, it is therefore important to assess participants in as realistic conditions as possible.

Even when on-road experiments are conducted, there are limitations to current approaches. For example, while Banks et al. (2018b) uncovered many usability issues with the Tesla automation, they did not probe drivers on their internal thoughts and could only infer user perceptions based on their recorded behavior. Endsley (2017) results came from a think-aloud protocol conducted on themself based on their *a priori* knowledge as a human factors researcher. While their results uncovered crucial issues concerning drivers' experience with a novel automated vehicle, we believe such a methodology is limited to explore how the different trust processes evolve to cope with risk while driving with automated capabilities. Critically, these studies have primarily focused on the behaviors, attitudes, and perceptions of the driver, but have not examined how trust evolves in groups of people while traveling together in AVs.

Building on this understanding, it is important to account for group factors and naturalistic social interactions when examining trust and risk in AVs (O'Malley et al., 1984; Van den Haak et al., 2004). Conventional methods such as the think-aloud protocol are limited because it can be unnatural to prompt participants (or prompt oneself, as was the case in Endsley, 2017) to divulge their inner thoughts while executing tasks (Jeffries et al., 1991; Nielsen, 1993; Douglas, 1995; Boren and Ramey, 2000; Smith and Dunckley, 2002). Constructive interaction, in which participants work together on tasks without persistent experimenter intervention, can mitigate these limitations. Constructive interaction relies on participants' natural tendency to reveal their thoughts, insights, and perspectives during the conversation, indirectly divulging their specific experiences, reasons, and decisions when interacting with technology (O'Malley et al., 1984; Nielsen, 1993; Douglas, 1995; Wildman, 1995; Kahler et al., 2000; Van den Haak et al., 2004). Indeed, an experienced researcher might not perceive an aspect of the task as risky, or cause for (dis)trust, enough to probe the user with the right questions. For novel users, this information is more likely to surface when interacting with other users.

### 1.4. Motivation and design of the current study

Hancock (2019) urges human factor researchers to consider the massive societal implications of AVs. The promise for AVs to transform society by reducing traffic deaths and improving mobility begets researchers to iron out current AV issues toward realizing this potential and foresee potentially new problems this new societal landscape may thrust upon us (de Winter, 2019; Emmenegger and Norman, 2019; Hancock, 2019; Waterson, 2019). To this end, the current experiment aimed to explore how trust processes unfold within groups in the face of a risky naturalistic setting. Participants, in groups of two or three, rode three loops in a Tesla Model X with Autopilot on campus roads while engaging in normal conversations. Conversations were then transcribed and analyzed for overarching themes in line with our research question:

How do group dynamics influence drivers' and passengers' trust processes in AVs?

Our research question was intentionally broad to allow for a wide-ranging exploration of themes that could be important in this naturalistic study. We hypothesized we would observe new insights into users' inner trust processes in a novel and potentially risky situation while driving or riding in an AV. This information will help to form the building blocks for a theory of how trust in automation processes evolves and interacts with group dynamics.

## 2. Methods

### 2.1. Participants

Twenty-four groups consisting of 65 participants (*M* = 24.23, age range: 18–52, 28 women) completed the experiment in exchange for course credit. The sample included six two-person groups and 18 three-person groups. Six groups were excluded from the analysis because their discussions were not recorded, or audio could not be transcribed. The remaining 18 groups consisting of four two-person groups and 14 three-person groups (*n* = 50 participants) were included in the qualitative analyses. All participants provided informed consent, and the research was approved by the Institutional Research Board at the United States Air Force Academy.

### 2.2. Task paradigm

**Figure 2:**
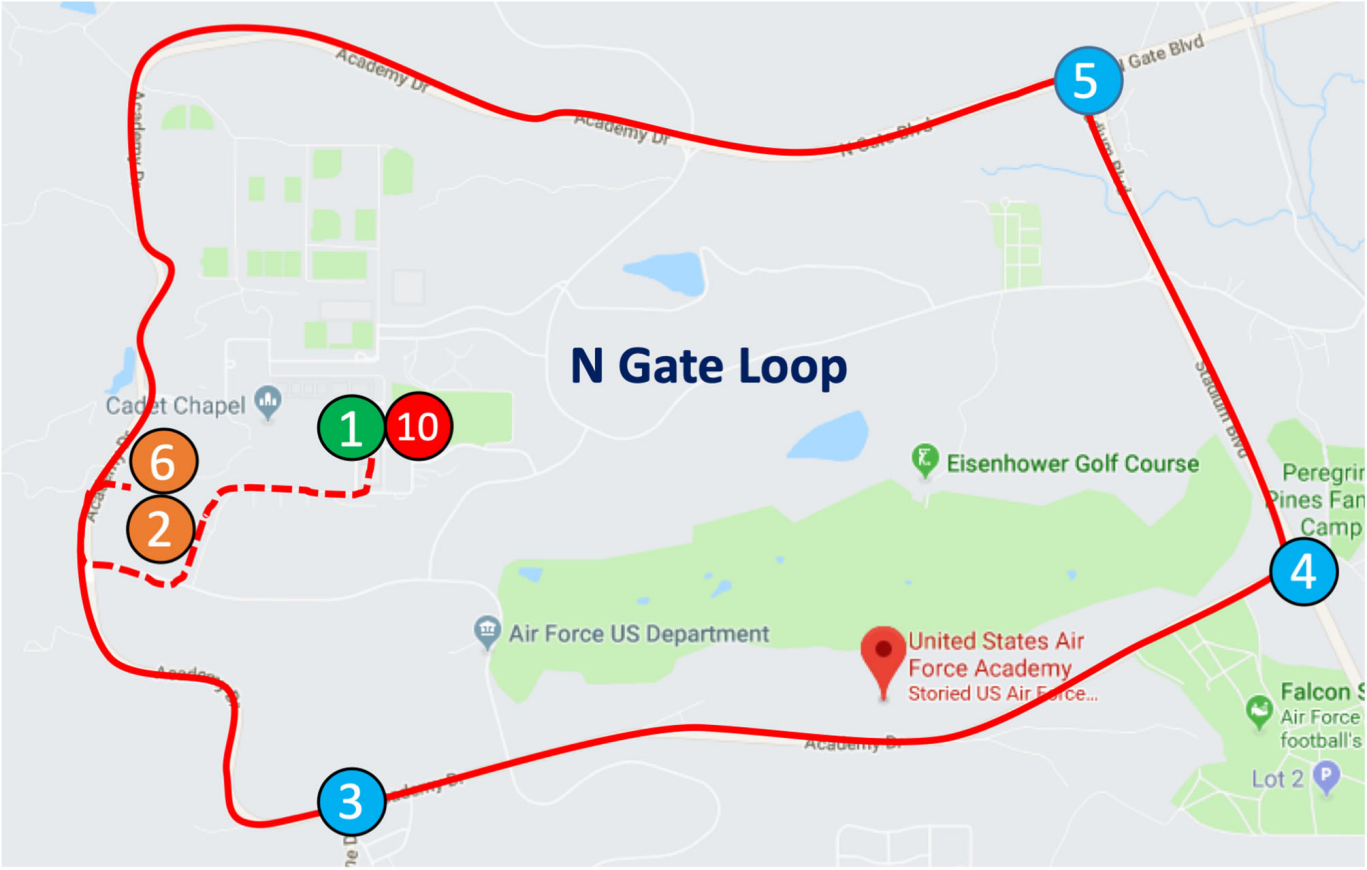
The USAFA loop. At the beginning of the experiment, participants exited the area at point 1 and began their driving loop from point 2 through point 6. At point 6, the car was stopped so participants could complete assessments. After the assessments on the first and second loops, participants turned left out of Point 6 and drove from point 2 through point 6. After completing the third loop, participants returned to point 7, instead of point 6, and concluded the experiment.

The driving task was performed using a 2017 Tesla Model X (Software Version 10.2) on the USAFA campus roadway. The driving loop took ~15 min, was 12.8 miles long, and consisted of two continuous segments of the road connected by two left turns at intersections with stop signs. Participants began at the Tesla's parking spot (see Figure 2, Point 1) and drove to Point 2 where they made a left turn onto the campus road, which began the “Loop.” To complete one full Loop the drivers continued driving down this road to (Point 3) and turned left. At the next intersection (Point 5), participants made a left and drove until they reached Point 6 (Campus Visitor Center), completing one loop. After completion of each of the first two loops, the driver was instructed to enter the Campus Visitor Center parking lot and park while riders completed the surveys. Afterward, the driver drove manually out of the parking lot and made a left onto the campus road toward Point 2, where the loop restarted. However, after the third and final loop, participants did not park at the visitor center and instead completed surveys back at Point 1, where the experiment concluded. During loops, participants were encouraged to engage in general conversation.

### 2.3. Automation driving behavior

Table 1 describes the automated features of Tesla's autopilot mode: traffic-aware cruise control (TACC), autosteer, automatic lane change, safety warnings, and disengaging autopilot (Autopilot, 2022). Participants were instructed to explore and use any of these features during the drive.

**Table 1:** Description of autopilot features.

**Tesla autopilot feature**	**Description**	**Maneuver**
Traffic-aware cruise-control (TACC)	The Traffic-aware cruise-control (TACC) senses other vehicles within its lane and slows down when vehicles get too close. The distance to the next car is determined by car lengths preferred by the driver (e.g., Tesla should stay four car lengths distant from the next car in front). During TACC, the Tesla generally drives at either the speed limit or the speed set by the driver	Drivers engaged TACC by pulling the automation stalk toward them, which was located on the bottom left of the steering wheel. TACC then showed an icon on the dashboard with the speed the Tesla is set at. The Tesla's speed could be adjusted by pushing down or up on the automation stalk. A single hard bump upwards increased the speed by 5 mph and a bump downwards decreased speed by 5 mph
Autosteer	Ability for the Tesla to stay within its lane when driving	To engage autosteer, drivers pulled the automation stalk toward them twice. Autosteer kept the vehicle within a traffic lane as well as utilizing TACC
Automatic lane change	Capability for the Tesla to execute a lane change by itself when safe	The automatic lane change feature engaged when autosteer was enabled and was triggered by the turn signal if Tesla detected the adjacent lane was not blocked by another vehicle
Safety warning	Occurred to remind drivers to keep their hands on the wheel if the Tesla was unable to sense the driver's hands during autosteer	The beep would continue to audibly escalate to remind the driver to apply force to the steering wheel. The Tesla automatically pulled over on the side of the road if the driver ignored these warnings. This did not occur for any groups in the experiment
Disengaging autopilot	Turning off autopilot	Pushing the automation stalk away from them, pressing the brake, or by applying force to the steering wheel

### 2.4. Procedure

Participants were first greeted by an experimenter who ensured all driving participants had a valid driver's license for a minimum of 2 years. The experimenter then obtained consent from all participants and chose at random which participants would be the driver, front passenger, and back passenger. Three-person groups filled out the vehicle in the driver, front passenger, and back right seats. Two-person groups filled out the vehicle in only the driver and front passenger seats. Participants then entered the vehicles at which point they were given a computer tablet to complete survey measures.

The driver was then trained and tested on the TACC, Autosteer, Automatic Lane Change, and Safety autopilot features of the car. The training session consisted of a 5-min training video created for this experiment (see Madison et al., 2021). The video trained the participant on how to safely operate the vehicle, obey the speed limit, activate and deactivate the autopilot features, safety warnings, and appropriate hand position and force on the steering wheel necessary to provide the Tesla feedback during autonomous mode. The training video was scripted primarily from Tesla's own descriptions of how to use its autonomous features (see https://www.tesla.com/support/autopilot) with some additional details, to ensure clarity and understandability of directions by the participants. Additionally, the driver was instructed to practice engaging and disengaging the features while they were briefed; the experimenter observed and corrected the driver if necessary. Once the driver felt comfortable and had asked any questions, the experimenter administered a post-training questionnaire to ensure the driver understood the Tesla's autonomous functions and could properly drive the vehicle. If the driver answered any questions incorrectly, the experimenter went through the training procedures again until they were sufficiently trained for the experiment.

Drivers were instructed to drive three loops with the automated driving functions as much as possible. The group was further instructed that during the drive they were to engage in “normal conversation.” Afterward, they were briefed on the directions of the driving task that they would begin at the Tesla's parking spot (Point 1) and drive to Points 2–6 to complete the loop three times. They were instructed to engage in normal conversation during loops and that after each loop they would fill out the quantitative measures. Specifically, after each of the first two loops, they would park at the Campus Visitor Center parking lot (Point 6) and park while the driver and passengers completed assessments. After the third and final loop, however, they would not stop at the visitor center and instead drive to point 1, where they began the experiment. Completing the assessments for the third and final time marked the end of the experiment. Participants were then debriefed and thanked for their participation. Safety was an emphasis of this training with explicit instructions that safety was the top priority of the study.

### 2.5. Data recording, reduction, and thematic analysis

Two GoPros recorded video and audio inside the vehicle. GoPros recorded the faces of the driver and front passenger as they watched the road through the windshield. We conducted a thematic analysis of these recorded conversations in the car in line with Braun and Clarke (2012). A thematic analysis uncovers overarching qualitative patterns defined as “themes.” This approach enabled insights into the novel construct of group trust dynamics in potentially risky scenarios.

#### 2.5.1. Phase 1: transcribing audio and identifying relevant conversations

Two researchers prepared the data for the thematic analysis. They began by extracting audio from the GoPro videos and having otter.ai (Otter.Ai—Voice Meeting Notes Real-time Transcription, 2022) automatically transcribe them. Annotators then watched the GoPro videos and from the transcripts identified relevant conversations pertaining to the Tesla and corrected grammatical inaccuracies. Conversations were defined as discussions among group members, regarding the Tesla or the drive, that differed across time and topic with at least one speech turn. Transcripts were sorted according to group, loop, and conversation numbers within each loop if multiple conversations occurred. We coded 97 conversations overall totaling 335 conversational turns (i.e., instances of alternating contributions and responses) between the participants (*mean conversation length* = 3.5 verbal exchanges).

#### 2.5.2. Phase 2: authors' review transcription

During the second phase, two of the authors independently reviewed the prepared transcriptions. Through multiple readings, authors took notes on a spreadsheet organized by conversation, loop, and group.

#### 2.5.3. Phase 3: initial coding based on notes

During the third phase, spreadsheets were combined with each author's notes and organized into their own column. The same two authors independently derived one to five initial codes per conversation per set of notes. Afterward, the authors came together and combined codes. Redundant codes were discarded. For example, “Team Mental Model Formation” was dropped as a unique code for being redundant with “*Group* Mental Model Formation.” Codes were combined when appropriate. At the end of this phase, there were a total of 78 codes and each code was supported by one to 44 pieces of information from the data.

#### 2.5.4. Phase 4: development of preliminary themes

During phase 4, the two authors worked together to identify patterns in the data representing potential higher-level themes. Together, authors uncovered themes and sub-themes by grouping codes that centered around similar relevant topics. For example, codes pertaining to “Emotion and Experience” were compiled, and from them derived sub-themes such as positive affect, negative affect, neutral affect, and risk and vulnerability. This process was repeated for all coding groups until all data was reviewed. This resulted in a preliminary list of five themes and 20 sub-themes.

#### 2.5.5. Phase 5: development of final themes

During the final phase, authors discussed and debated whether any themes or sub-themes should be combined, discarded, or disbanded and rearranged, paying close attention to whether they addressed the research question. For example, the previous theme of “Emotion/Experience” was combined with “Group Emotions” to form the new overarching theme of Collective Risk Perception. The neutral affect sub-theme was discarded. At the end of this phase, there were a total of five themes. After the final themes were decided, all conversations were recorded and consolidated into these codes (see Table 2).

**Table 2:** Themes with description, trust process, number of conversations, number of groups, and percentage of groups.

**Themes**	**Description**	**Trust process**	**#C**	**#G**	**%G**
Collective risk perception	*Tesla's autopilot leads to feelings of vulnerability and high risk*	Affective	56	17	94
Experimenting with automation	*Drivers test whether the Tesla autopilot can handle certain traffic situations*	Analog	18	10	56
Group sense-making of automation	*Shared cognitions and attitudes about automation among group members of a system*	Analog	57	17	94
Human-automation interaction issues	*Design issues with the automation*	Analytic	17	8	44
Automation benefits	*Benefits of driving with automation*	Analytic	21	12	67

For each sub-theme, the number of conversations (#C) and groups (#G) that mention it are calculated along with a group representativeness percentage (%G) calculated by dividing the number of groups that mentioned the theme by the total number of groups.

## 3. Results and discussion

Results were structured in the following section by themes and associated sub-themes (see Table 2). Quotes are cited with the convention of G = group number, L = loop number, and C = conversation number (see Table 2). Quotes also denote group member source as either the driver, the front passenger (Front), or the back passenger (Back).

### 3.1. Collective risk perception

Seventeen groups (94%) had 56 conversations related to collective risk. Groups experienced risk and vulnerability during autopilot that typically elicited negative emotions that tended to be shared among the group. Participants felt a heightened sense of risk during specific events such as sharp turns and curvy roads (*G3, L1, C2; Front: Was [the autopilot on] through that twisty area? Driver: No, but if I had reduced the speed limit, I would have been okay*), and the Tesla accelerating in response to changing roadway conditions:

*G5, L1, C9; Driver: So now I feel like I'm going too fast, I'm catching up to everyone, but I know the speed limit was 55 here*), another vehicle cutting in front (*G14, L1, C2; Driver: when that car turned in front of us, I was like “I don't wanna die I don't want to hurt the car!”*

**Figure 3:**
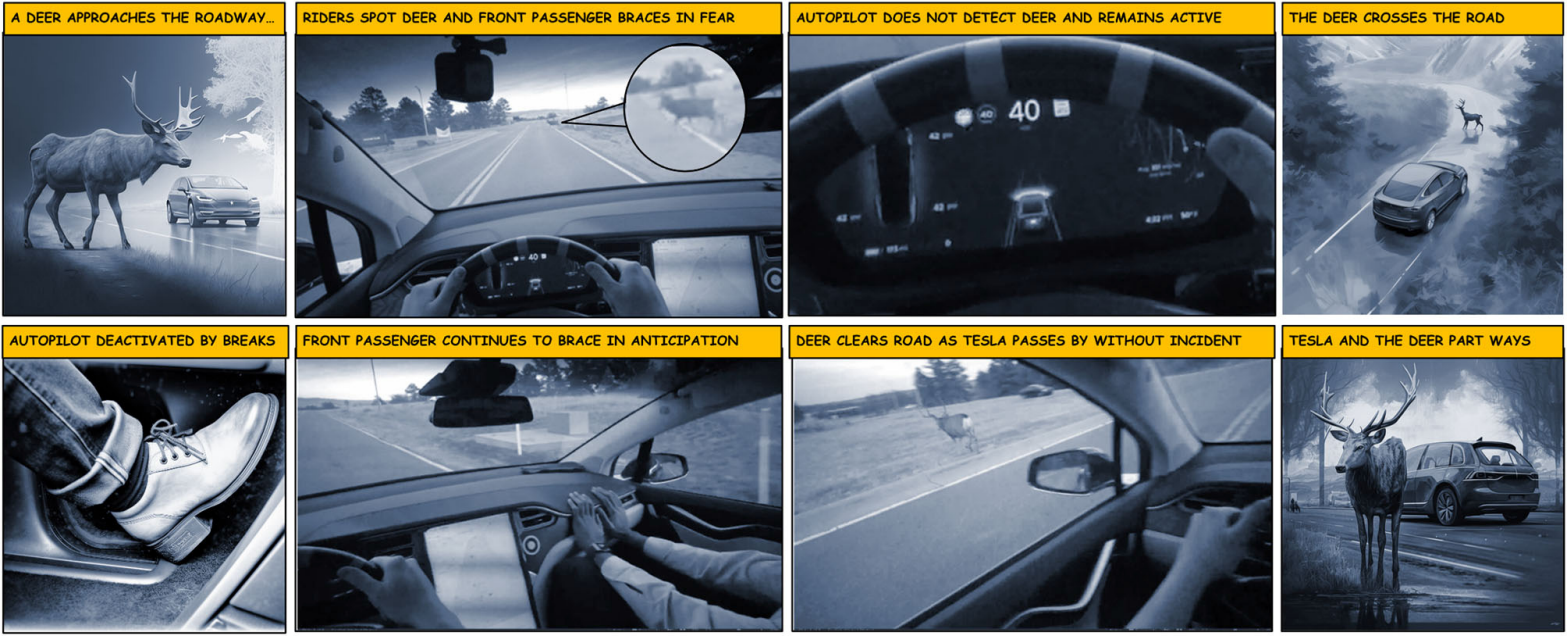
Sequence of events while encountering a deer on the road while driving on autopilot. The middle four images are recorded videos from the driver's perspective of the experimental driving session for Group 6. The outer four images were all AI-generated images by Midjourney to better contextualize the video footage (Midjourney Documentation User Guide, 2023).

Finally, participants felt heightened risk during unexpected events on the road. In an example of an unexpected roadway event, a deer crossed the road while the Tesla was on autopilot (see Figure 3). The driver waited for an automatic stop to happen, which never occurred, before taking control of the vehicle at the last minute, causing distress to the passengers. In this case, negative emotions even lingered into later parts of the drive, such as when the passenger sarcastically told the driver not to miss a stop sign (*G6, L3, C5; Front: I can tell you haven't drove since you've been here*). Negative emotions also served as points of conflict when there was disagreement regarding the perceived lack of control experienced with the Tesla:

*G14, L2, C2; Driver: I feel like autopilot is pretty convenient. I don't think about it. Front: I feel like I wouldn't want autopilot on my vehicle. I like to observe things and do everything myself* .

Our findings demonstrate riders experienced risk often and collectively, leading to negative emotions that were experienced by the entire group, consistent with the *affect heuristic* (Loewenstein et al., 2001; Slovic et al., 2007). Given that risk perception plays prominently in the user's trust perception in the form of an *affective trust process* (Lee and See, 2004), it is possible that this would increase a rider's distrust in Tesla, leading to disuse (Hu et al., 2022). This is consistent with previous studies which assert that risk and vulnerability is the *key* factor in the development of trust and should be induced for a realistic assessment of trust (Wagner et al., 2018; Wagner, 2020). It is unclear whether the collective experience of emotion is fully in response to Tesla's performance or also partly in response to the spreading of negative emotion among riders, a phenomenon known as *contagion* (Loersch et al., 2008). Goldenberg and Gross (2020) assert that digital networks, such as social media, cause our own emotions to become more like others, and is heightened due to increased exposure to these technologies. It is unclear if rider distrust is a result of technology-driven contagion, social-group-driven contagion, or a combination of both.

### 3.2. Experimenting with automation

**Figure 4:**
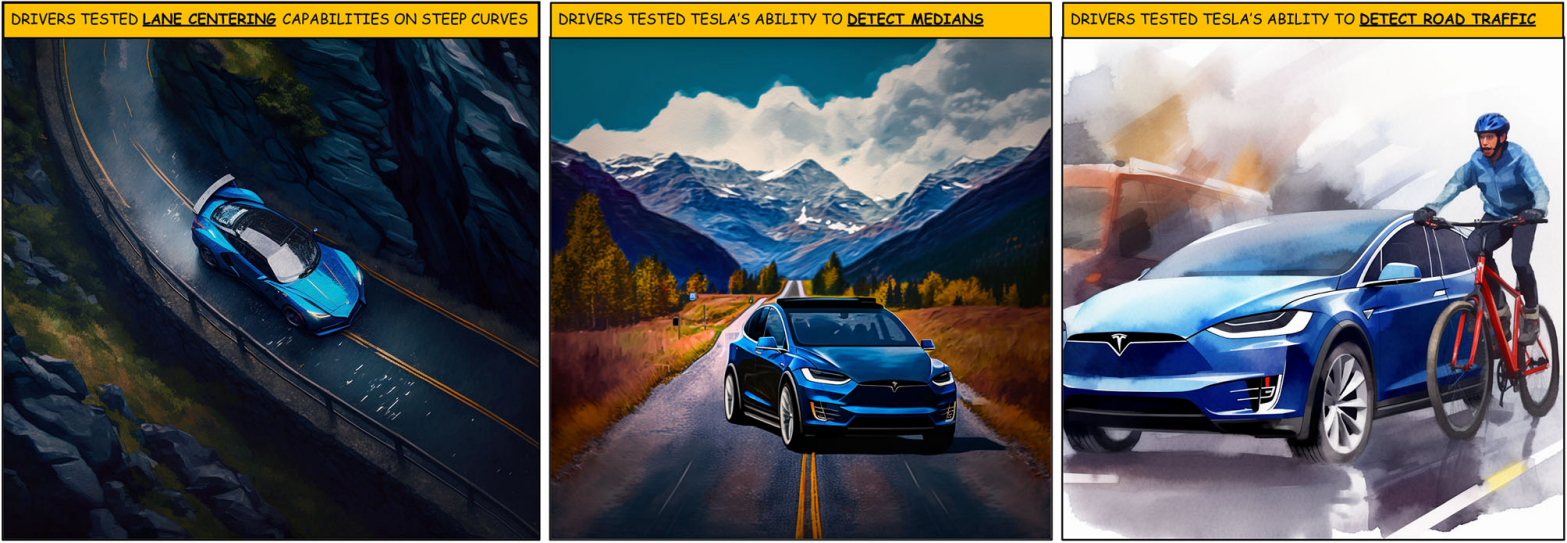
Examples of scenarios where drivers experimented with Tesla's abilities. All images were AI generated by Midjourney (Midjourney Documentation User Guide, 2023).

Ten groups (56%) had 18 conversations pertaining to experimentation with the autopilot features (see Figure 4). Groups experimented with automation to figure out how it worked. In one of these instances, a driver tested the autopilot's lane following during a sharp turn in which a vehicle was passing in the opposite lane:

*G7, L2, C1; Driver*: …*but, instead of just grabbing [the wheel], I was ready to stop [the wheel] if it kept turning. And what it did was, it just straightened out in the center of the [lane]*.

Another group tested whether the autopilot would detect a median, finding the Tesla swerved before reaching the median to follow the lane (*G14, L2, C3; Driver: I wonder if it knows there's a median, we should look into that... It made me turn! I think the steering moved for me*). Another group tested whether the autopilot detected a bicyclist in the adjacent lane:

*G1, L3, C1; Driver: Okay. What does the car do with a cyclist? Okay good to know. Passenger: What happened there? Was it trying to correct back into the cyclist? Driver: At least I did not detect a recognition of the cyclist. I went left across the dividing line and the cyclists did not appear on the little display. So, I got worried that this thing wasn't going to actually correct for the cyclist, so I intervened*.

Experimenting with automation can be classified as an *analogical trust process* of direct observation and interaction with the agent (Lee and See, 2004; Hoff and Bashir, 2015). Two examples of a similar theme of “Testing the Limits of ODD (Operational Design Domain)” was observed in Banks's et al. (2018b) Tesla self-driving study. In their prototype example, a driver similarly experiments with the autopilot's lane-following, while maintaining control, by releasing and hovering their hands over the wheel. The authors attribute this as risky behavior stemming from a “natural curiosity” to test a machine's limits. However, we demonstrate, in many more instances, that these risky experiments may also be a mechanism for the driver and group to collectively calibrate their trust in the Tesla. As we demonstrate, the driver is learning out loud, enabling other group members insight into the driver's first-hand knowledge. Experimentation within a group setting like this may be further explained by a common phenomenon in human–human groups to *polarize*, or, make more extreme decisions than individuals (Moscovici and Zavalloni, 1969; Fraser et al., 1971; Myers and Lamm, 1976). Groups make a “risky shift,” or accept more risk, when the group perceives risk as positive but a “cautious shift” when they perceive caution as negative (Wallach et al., 1962). Further research has found that this effect extends to technology, with groups' trust in technology as more extreme (greater trust or greater distrust) than individual trust (Xu et al., 2014; Martinez et al., 2023).

### 3.3. Group sense-making of automation

**Figure 5:**
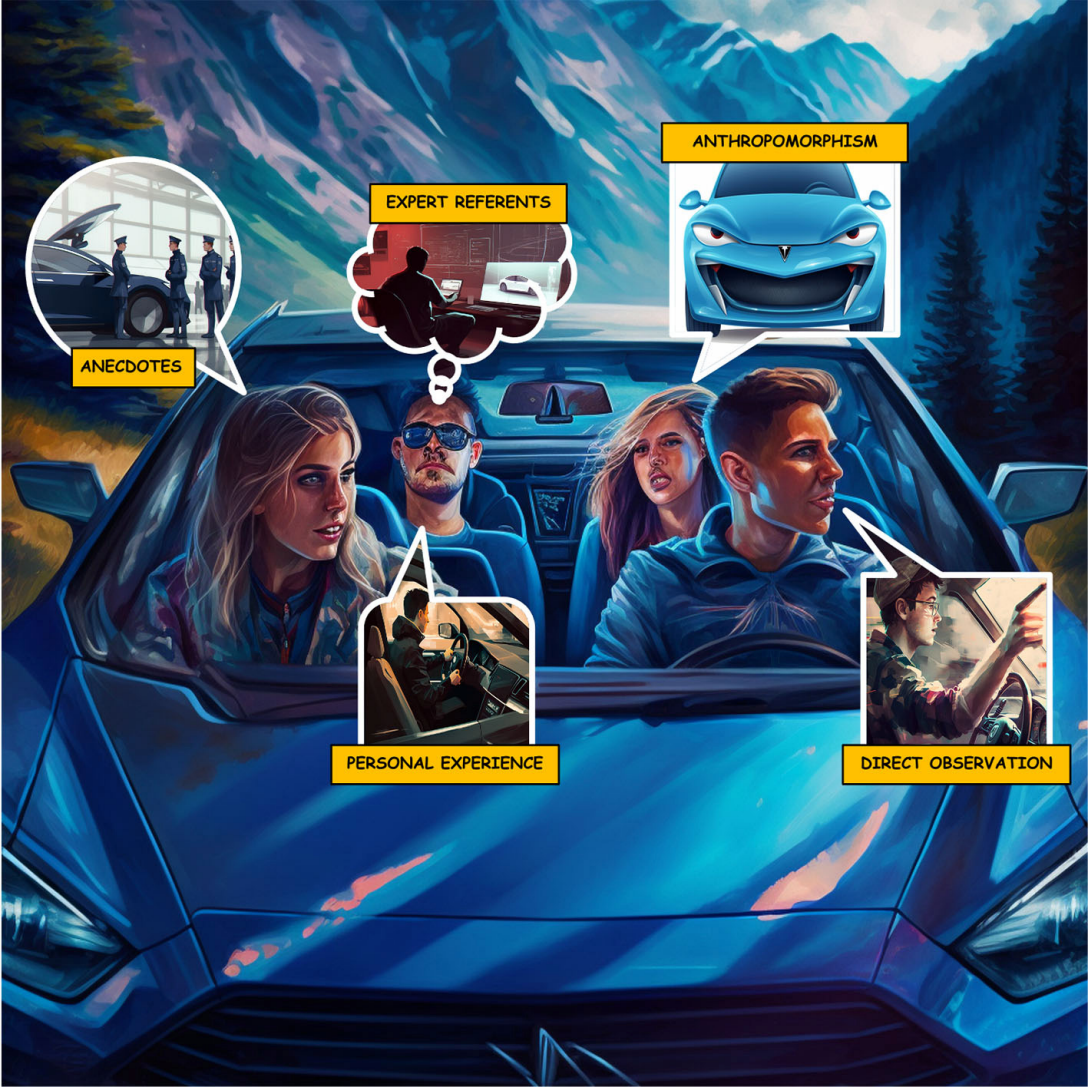
Five sources of information that groups used to make sense of automation. All images were AI generated by Midjourney and then compiled and labeled in this collage (Midjourney Documentation User Guide, 2023).

Seventeen groups (94%) had 57 conversations pertaining to group sense-making with the autopilot features (see Figure 5). Groups used several sources of information to better understand and predict automated behavior via sharing (1) direct observations about the Tesla autopilot, (2) expert referents, or, sources of credibility and expertise from people, media, or other technology, used to help understand automated driving technology, (3) anecdotal stories, and (4) anthropomorphism. When participants shared information directly observed about the Tesla autopilot, it was typically either (1) offered unsolicited by the driver or front passenger to help the other riders better understand the experience:

*G14, L1, C1; Front: Oh yeah that was cool. Right when we passed that [40 mph speed limit] sign it changed. Driver: It doesn't change to 40? Front: ...because you set the max, it just tells you the speed limit*.

or (2) elicited by the front passenger asking the driver a question:

*G1, L2, C3; Front: Was that turn all the car? Was your hand just lightly on the wheel but it was doing the turning? Driver: Yeah. I let it do that whole turn, but it didn't quite stay in the lines*.

The backseat passenger seldom contributed information. For the second information source, expert referents typically served as first impressions of the Tesla's automation capabilities and were made of family members (*G1, L2, C2; Passenger: my son has a Tesla. He loves it. He's uh, you know, a techie engineer guy*), their own vehicles (G1, L2, C2; D*river: wish I could engage an autopilot on my Honda Odyssey because you are on a freeway, straight, can see for infinite ahead and think “why in the world am I driving this car under these circumstances”*) technology in general, social media, and pop culture reporters:

*G1, L3, C2; Driver: About three weeks ago, Malcolm Gladwell put out one of his revisionist history podcasts. He and his producer went down to Phoenix and messed around with Waymo for a day, and that was pretty interesting. There are Waymos just driving around, you can order one up like an Uber. And it's a Chrysler Pacifica and with a bunch of sensors on top, no engineer, no person in the car, just the car and it comes and gets you and drives you to your destination. You ride in the back*.

For the third information source, groups often told anecdotes and stories when communicating about their own and other vehicles (*G3, L1, C2; Driver: It's a feature, car is locked, you can't open the gas cap door, that's good*), educational videos on social media, and other Tesla's (*G9, L1, C1; Driver: one time my friend's dad picked me up in their Tesla from school and, from the way it decelerated, I thought I was gonna throw up after that*). For the fourth information source, anthropomorphism occurred when participants named the car (*G9, L2, C2; Front: We like to name our cars. So, we ended up naming this one Karen, because it would constantly beep at you. Constantly*) in response to negative emotions regarding the audio warnings, and to show affection (*G9, L2, C2; Front: My folks got a Kia Telluride. I was trying to think of an affectionate name for it. Like “Telly” or “Tia”)*.

Our results agree with several areas of literature associated with groups and sense-making and fits with the *analogical trust process* of reputation and gossip (Lee and See, 2004). Riders sharing information about their driving experience falls in line with Bolstad and Endsley's (1999) principles of team mental models where (1) group members share pertinent information with each other, (2) communicate verbally and through technological mediums, (3) have accurate shared mental models, and (4) and engage in effective collaboration and planning. Indeed, riders shared pertinent information, communicated, and, at times, had accurate mental models. Featured prominently in our theme of group sense-making is that groups often shared stories of previous experiences to make sense of the novel Tesla autopilot. Indeed, storytelling is an oft-cited way for groups to deal with perceptions of risk in novel or uncertain situations (Bietti et al., 2019). Given that risk shapes perceptions of trust, this also connects with recent automation literature in trust propagation. Trust propagation occurs when an individual's perception of trust is shaped indirectly through the direct experience of another individual. Our results add to the literature by findings trust propagation occurs frequently in AVs, likely due to the risk participants faced by our naturalistic design. The extent that storytelling is an effective method to alleviate uncertainty in an AV should be further examined in future research. The utilization of expert referents to inform trust assessments is also consistent with findings on the concept of trust propagation (Guo et al., 2023). In this case, trust referents came from family members, pop culture reporters, experience from their own vehicles and technologies in general, and social media. A similar mechanism of peer influence was observed with parents' willingness to let their children ride unattended in an AV (Ayala et al., 2022). Users are also anthropomorphized by conversing and relating their experience with other vehicles to their experience with the Tesla. Epley et al. (2007) suggests individuals default to anthropomorphizing technology because they lack knowledge about the internal workings of technology yet have a vast understanding of human-like traits readily available. This tendency to anthropomorphize is heightened by effectance motivation—the desire to interact effectively with the environment—and sociality motivation—the desire for social connection. When riders named the vehicle “Karen” due to its unpredictable and constant beeping, it was likely to satisfy effectance motivation. Indeed, a commonly observed phenomenon is to name natural disasters, which are also unpredictable and distressing (Storms Payback from God, Nagin Says Mayor Faults War, Blacks' Infighting—The Washington Post, 2006). When a rider named the vehicle “Telly” to show affection, it was likely out of sociality motivation. Indeed, research has shown that naming non-human agents, such as technological gadgets, can satisfy our need for social connection (Epley et al., 2008a,b; Powers et al., 2014).

### 3.4. Human-automation interaction issues

**Figure 6:**
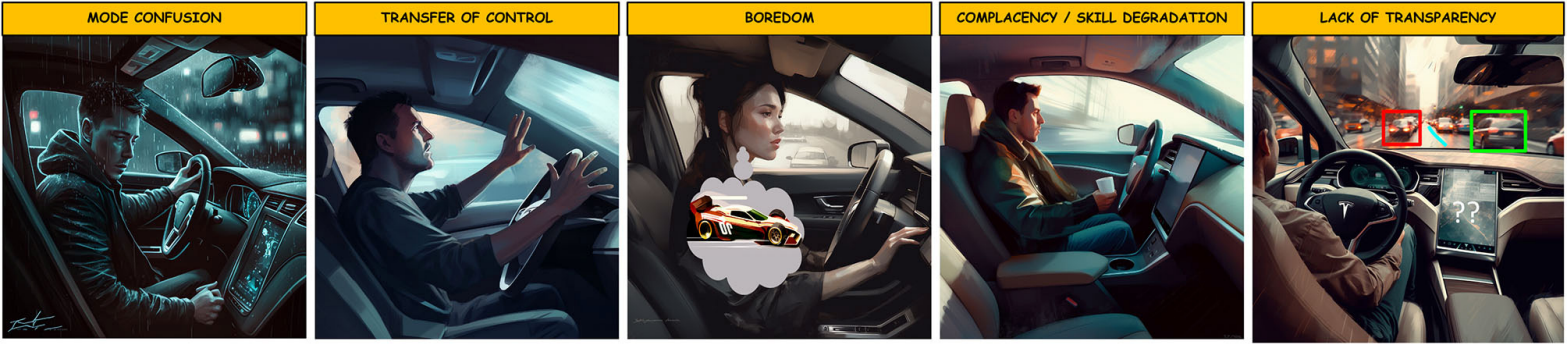
Human-automation interaction issues were experienced and discussed in the groups during the drives. All images were AI generated by Midjourney (Midjourney Documentation User Guide, 2023).

Eight groups (44%) had 17 conversations identifying several human-automation design issues with the Tesla automation (see Figure 6), following an *analytical trust process* (Lee and See, 2004). These included issues with the autopilot features such as mode confusion—confusion over the on/off status of autopilot:

*G3, L1, C2; Experimenter: Yeah, go ahead and turn the autopilot back on. Driver: Oh, it's [autopilot] not on? I thought it was on. Front: Okay, so you were just going, that was autopilot through that twisty area? Driver: No, I think I turned it off. Yeah*.

Mode confusion was found in previous experiments examining driver behavior in Tesla (Nikolic et al., 1998; Endsley, 2017; Banks et al., 2018a). Drivers were unaware the autopilot had turned off is a potential safety issue as this occurred during critical times in the drive. Banks et al. (2018a) posits this occurs because the autopilot status directs the driver's attention to outside the vehicle rather than the controls inside the vehicle while Endsley (2017) cites a lack of salient audio warnings. The group nature of our experiment, we believe, also contributed to mode confusion by passengers serving as an additional distraction (Laberge et al., 2004; Neyens and Boyle, 2008; Zhang et al., 2019). A second issue was the transfer of control—the action of transferring between autopilot and manual features—engaging autopilot via the automation lever, deactivating autopilot with the steering wheel, and utilizing automatic lane change (*G10, L1, C5; Driver: I don't have another lane to try [automatic lane change]...I feel like that's asking for a problem*). Previous research demonstrates that trust during the transfer of control maneuver is related to the comfort of riders in simulated driving tasks (Molnar et al., 2018; Petersen et al., 2019). Given our naturalistic experiment on public roads, it is likely to transfer of control issues further exacerbated riders' perceptions of comfort during the drive. Together, these findings underscore the importance of smoothly transferring control from manual to autopilot. Another issue pertained to boredom resulting from the lack of tasks and engagement when the autopilot was on (*G14, L2, C2; Driver: I feel like I wouldn't want autopilot on my vehicle. I like to observe things. I would rather be doing everything myself*). The boredom experienced by drivers during the experiment could also be a potential safety issue. Indeed, vigilance research demonstrates that attention can be sustained for so long before decrements in performance occur. Similarly, transportation research demonstrates prolonged exposure to highly automated driving can lead to driver disengagement (Stanton and Barnes-Farrell, 1996; Young and Stanton, 2002; Saxby et al., 2007; Endsley, 2017) and automation complacency (Parasuraman et al., 1993; Lee and See, 2004; Hollnagel and Woods, 2005). If drivers do not appropriately monitor Tesla, it would be potentially unsafe. It is important for automated driving systems to ensure drivers are actively and positively engaged. A fourth issue pertained to skill degradation, in which drivers mentioned a lack of motivation to go back to standard driving, and the lack of involvement of feet during automated driving (*G9, L2, C3; Driver: I'm still afraid that driving regular would feel pretty boring because this is really like trying to match my correct speed knowledge*). Given that roadways are composed of mixed traffic—both non-automated and automated vehicles—the potential for AV drivers to be unsafe when going back to normal vehicles having lost essential driving skills is an issue that could occur with ubiquitous and long-term automated driving (Parasuraman et al., 2000; Saffarian et al., 2012). A fifth issue groups mentioned regarding Tesla was its transparency. One group was unclear regarding the Tesla's process of detecting events (i.e., speed limit sign) via its sensors and that it sometimes was unclear how the Tesla made an error (*G1, L1, C1; Driver: I wonder why it thinks that's that speed limit... I don't know what baseline map Tesla uses*). One group conversed about why Tesla's process for projecting roadway events and why it did not detect a cyclist:

*G7, L3, C1* Driver: *When we had that Jeep in front of us on the other road [we got] a little picture. But the cyclists did not appear on the little display, so I got worried that this thing wasn't going to actually correct for the cyclist*.

Additionally, groups noted how the Tesla communicated via warning beeps as well as failures to communicate information (*G24, L3, C1; Front: I did hear it go beep. I'm not sure if it's because you hit the brake or if it actually saw the deer*). The need for increased transparency into Tesla's autopilot behavior is in line with other findings (Banks and Stanton, 2016; Banks et al., 2018a; Chen et al., 2018). Thus, it is key that AV designers focus on displays that are consistent, provide accurate information and convey uncertainty when sensors are unreliable or unavailable (Kraus et al., 2020). Additionally, designers should expand on the capabilities of the display. Endsley (2017, 2023) similarly found that, while Tesla's display increased transparency, it failed to provide insight into its internal workings. Designing Tesla displays to visually indicate its current range of detection, information showing its decision-making, and confidence would greatly improve riders' experience and ultimately safe use of the AV (Du et al., 2019; Maarten Schraagen et al., 2021).

### 3.5. Automation benefits

**Figure 7:**
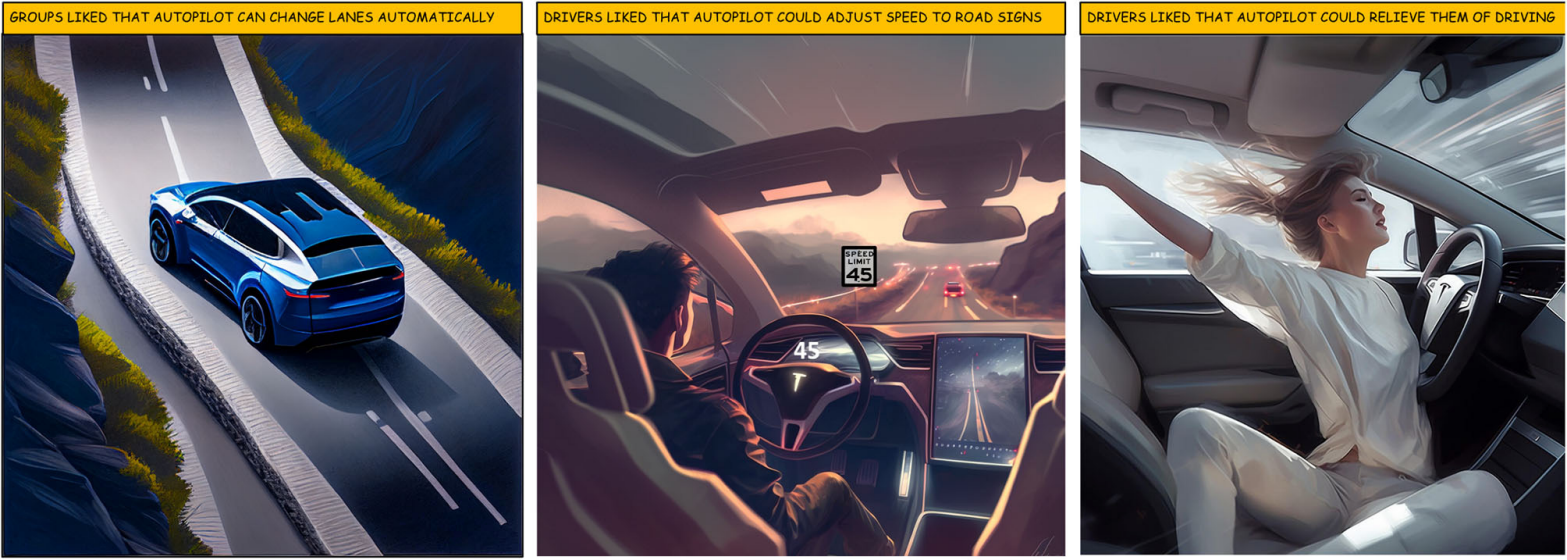
Three benefits of driving with automation. All images were AI generated by Midjourney (Midjourney Documentation User Guide, 2023).

Twelve groups (67%) had 21 conversations pertaining to the benefits of driving with automation (see Figure 7). Aspects of the Tesla's autopilot regarded as particularly positive were when it acted reliably during autopilot, such as lane-centering, adaptive cruise-control, automatic lane change, auto-breaking, and the perceived lack of work required during autopilot. One group liked that the Tesla could perform a lane change *(G2, L3, C1 Driver: [Completes automatic lane change] wow, that's cool)*. Groups also liked when the automation accurately provided transparency into the system. Specifically, they noted the Tesla's detection of events via its sensors such as when it detected changes in speed limits, and roadway events such as registering objects or other inhabitants of the road (*G16, L2, C3; Driver: this is crazy that a car can even do something like this but there's some smart people to figure out*).

Identifying automation benefits constitutes an analytical trust process (Lee and See, 2004). Previous research also demonstrates drivers positively regard autopilot features during ordinary parts of the drive (Endsley, 2017; Banks et al., 2018a). These positive benefits of autopilot speak to the promise of automated vehicles. On the other hand, Banks et al. (2018a) found drivers felt positive to the extent they released their hands from the wheel and engaged in non-driving secondary tasks until prompted by the driver monitoring system. This is a potential safety concern if drivers become too comfortable and disengage from the driving task and the Tesla's autopilot suddenly becomes unreliable.

### 3.6. Revised group trust process model

**Figure 8:**
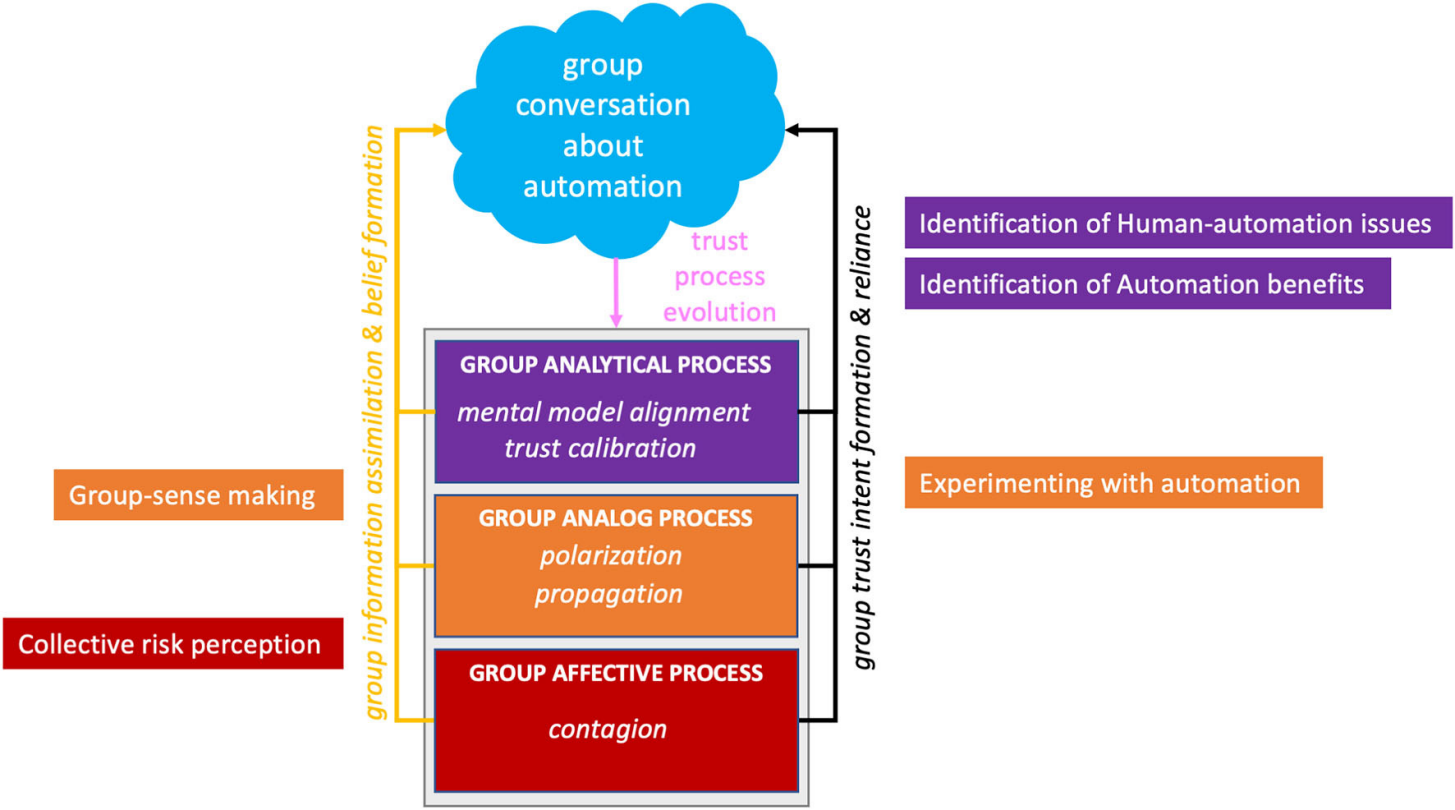
Revised Lee and See's (2004) model to capture trust processes as part of group dynamics.

We have further determined where the five themes fit into Lee and See's (2004, p. 54) expanded model (see Figure 8). The model is divided into four stages, including (1) information assimilation and belief formation, (2) trust evolution, (3) intention formation, and (4) reliance action, which is reproduced in our model (see Figure 8). Given this taxonomy, we can group the themes group sense-making and collective risk perception into stage 1 since these themes involve the processing of information and beliefs. The human-automation issues and automation benefits can be classified into stage 3 because these are specific attitudes toward automation. Experimenting with automation involves stage 4 because riders are actively figuring out their reliance strategy in real time.

## 4. General discussion

### 4.1. Trust process mechanisms in groups and teams: a revised model

The purpose of our study was to uncover how group dynamics influenced trust processes while interacting with and traveling in an automated vehicle. Our experiment allowed us to explore how trust processes unfold within groups in the face of a risky naturalistic setting. Our five themes aligned with Lee and See's (2004) analytical, analog, and affective processes of trust evolution and formation (see Figure 8). Specifically, our themes of automation benefits and human-automation interaction highlight the analytical nature of trust. Indeed, riders cognitively reasoned and came to judgments regarding certain aspects of Tesla's capabilities (i.e., automation benefits) and deficiencies (i.e., human-automation interaction). These judgments were shared extensively by riders through conversation, potentially through candidate group mechanisms such as shared mental models and/or a trust calibration in the automation process occurring collectively among the group. Our themes of experimenting with automation and group-sense making highlight the analogical nature of trust. Candidate group mechanisms for this process likely include polarization and propagation. Indeed, experimenting with automation and the direct observation source of group-sense making both leverage direct observations of Tesla's behavior in response to roadway conditions. Additionally, the expert referents aspect of group-sense making is in line with reputation from the analog trust influence while the anecdotal stories facet of group-sense making connect with the analog source of information stemming from reports from parties that have experience with the agent. More broadly, influences of expert-referents and anecdotal stories bear out Lee and See's (2004) proposed influence of the organizational context of trust. When individuals lack information, the organization can serve as a source of information through gossip (e.g., anecdotal stories) and reputation (e.g., expert referents). Finally, our theme of collective risk perception highlights the affective nature of trust, as negative attitudes stemming from perceived risk were typically rapidly and reactively experienced by the entire group on short-time scales. Contagion is a likely candidate mechanism for this effect. For all identified trust and group processes and effects discussed here, we emphasize these are hypothesized as they arose from our naturalistic AV setting. The group's processes revealed here confirm the importance of the social and relational nature of trust and should be incorporated into theories of trust (de Visser et al., 2018; Chiou and Lee, 2021). Developing a group process theory of trust in automation will require further testing and validation in more rigorous experimental settings.

### 4.2. Practical implications for improving AV technology

One way to calibrate trust in the face of risk, uncertainty and human automation issues (i.e., mode confusion, transfer of control, boredom) may be through effective instructional strategies (Dikmen and Burns, 2017; Endsley, 2017). Dikmen and Burns (2017) and Endsley (2017) similarly acknowledge the necessity of instructional videos that demonstrate the system's capabilities and could be hosted online and continually updated. Beyond demonstrating the system's capabilities, our results further suggest that training should demonstrate driving automation's in-the-wild behavior during uncertain, rare, and hazardous traffic scenarios. In those circumstances, our groups most often experienced risk and experimented with AV. Future experiments should explore the most effective instructional strategies to calibrate trust with an AV so that people who over-trust, adjust their trust downward and people that distrust, adjust their trust upward (Parasuraman et al., 2014; Forster et al., 2018; Azevedo-Sa et al., 2020; de Visser et al., 2020). Ultimately, such “trust in automation” training could be delivered when AVs are delivered to their new owners or as part of a requirement to obtain special AV driving licenses (Cummings, 2019). In addition to training, some of the observed human automation issues could be resolved through improved design. Mode (automation vs. manual) and transfer of control is currently indicated by visual and audio cues that lack appropriate salience. Future designs could incorporate larger visual cues and improved audio alerts that better alert the driver. As participants often felt distracted by jarring loud beeps, it is further important that audio alerts sound pleasing, which may be achieved through musical sonification (Gang et al., 2018; Seppelt and Lee, 2019; Chen and Chen, 2021; Nadri et al., 2023). Aside from multimodal cues, we recommend a different transfer of control maneuver (e.g., button) that is more straightforward than the current lever maneuver. Issues with transparency into the system's performance via the display likely contributed to collective perceptions of risk and uncertainty. As stated before, Tesla displays should better indicate its current range of detection and incorporate information about its decision-making and confidence (Maarten Schraagen et al., 2021; Endsley, 2023). Finally, unobtrusive physiological measurements of both drivers' and passengers' cognitive state and trust could provide critical data to improve AV safety (Tenhundfeld et al., 2019; Belcher et al., 2022).

## 5. Conclusion

In conclusion, our research underscores the importance of understanding trust within group contexts and its implications for the development and acceptance of AV technology. Our findings align with Lee and See's (2004) trust process model, shedding light on the analytical, analogical, and affective nature of trust in AV systems. We extend this study by showing that conversations among group members are significant to consider as group mechanisms such as shared mental models, polarization, propagation, and contagion factor into trust perceptions and decision-making. More practically, our study has implications for improving AV technology for enhanced trust calibration. Effective instruction and design elements, including more salient visual and audio cues, hold promise for enhancing human-automation trust. Importantly, our study highlights the crucial role of studying trust in more ecologically valid environments and the role of group processes in the development of trust in AVs.

## Data availability statement

The raw data supporting the conclusions of this article will be made available by the authors, without undue reservation.

## Ethics statement

The studies involving human participants were reviewed and approved by Institutional Research Board at the United States Air Force Academy. The patients/participants provided their written informed consent to participate in this study.

## Author contributions

MF conceptualized the study. AMo collected the data and analyzed the data. KC, EV, and AMo analyzed the data. EV, AMa, CT, KC, and AMo interpreted the results and wrote the manuscript. All authors contributed to the article and approved the submitted version.
